# Multi-label Deep Learning for Gene Function Annotation in Cancer Pathways

**DOI:** 10.1038/s41598-017-17842-9

**Published:** 2018-01-10

**Authors:** Renchu Guan, Xu Wang, Mary Qu Yang, Yu Zhang, Fengfeng Zhou, Chen Yang, Yanchun Liang

**Affiliations:** 1 Key Laboratory for Symbol Computation and Knowledge Engineering of National Education Ministry, College of Computer Science and Technology, Jilin University, Changchun, 130012 China; 2 Zhuhai Laboratory of Key Laboratory of Symbolic Computation and Knowledge Engineering of Ministry of Education, Zhuhai College of Jilin University, Zhuhai, 519041 China; 3 MidSouth Bioinformatics Center and Joint Bioinformatics Ph.D. Program of University of Arkansas at Little Rock and Univ. of Arkansas Medical Sciences, Little Rock, AR 72204 USA; 4 Institute of Information Engineering, Chinese Academy of Sciences School of Cyber Security, University of Chinese Academy of Sciences, Beijing, 100093 China; 5 College of Earth Sciences, Jilin University, Changchun, 130061 China

## Abstract

The war on cancer is progressing globally but slowly as researchers around the world continue to seek and discover more innovative and effective ways of curing this catastrophic disease. Organizing biological information, representing it, and making it accessible, or biocuration, is an important aspect of biomedical research and discovery. However, because maintaining sophisticated biocuration is highly resource dependent, it continues to lag behind the continually being generated biomedical data. Another critical aspect of cancer research, pathway analysis, has proven to be an efficient method for gaining insight into the underlying biology associated with cancer. We propose a deep-learning-based model, Stacked Denoising Autoencoder Multi-Label Learning (SdaMLL), for facilitating gene multi-function discovery and pathway completion. SdaMLL can capture intermediate representations robust to partial corruption of the input pattern and generate low-dimensional codes superior to conditional dimension reduction tools. Experimental results indicate that SdaMLL outperforms existing classical multi-label algorithms. Moreover, we found some gene functions, such as Fused in Sarcoma (FUS, which may be part of transcriptional misregulation in cancer) and p27 (which we expect will become a member viral carcinogenesis), that can be used to complete the related pathways. We provide a visual tool (https://www.keaml.cn/gpvisual) to view the new gene functions in cancer pathways.

## Introduction

Cancer research has witnessed rapid advances year by year, generating a more abundant and complex body of knowledge. Researchers continue to come up with ingenious approaches for treating, preventing, and curing the disease. However, the war on cancer still has a long way to go^1^. Among the 22 novel drugs approved by the U.S. Food and Drug Administration (FDA), six of them were designed for treating or diagnosing cancer^2^. Tomas Lindahl and Paul Modrich’s Mechanistic Studies of DNA Repair won the 2015 Nobel Prize in Chemistry, and their work may potentially advance the development of new cancer treatment^3^. In 2016, the 21st Century Cures Act provided 4.8 billion for the Cancer Moonshot^4^ and Precision Medicine Initiative, which aims at dramatically accelerating efforts to prevent, diagnose, and treat cancer^5^. Scientists from various fields, such as biology, statistics, and computer science, are using a vast array of approaches, trying their best to not only wage a battle but win the war against cancer worldwide.

Among these approaches, biocuration, which involves organizing, representing, and providing biological information for humans and computers, is an essential part of biomedical discovery and research^6^. At the present rate, however, the further and farther the curated data lags behind current biological knowledge, either way, the greater and more apparent the daily knowledge gaps will become. Originating with the Human Genome Project (HGP), microarray expression analysis, investments in large-scale sequencing centres and high-throughput analytical facilities have been increasing sharply, all leading to the exponential growth of biological data. The 2016 Nucleic Acids Research (NAR) online database collections, containing 15 categories and 41 subcategories, listed 1664 published biological databases. By July 2017, more than 27 million citations for biomedical literature from MEDLINE, life science journals, and online books had been indexed in Pubmed^7^. However, the resources for generating and testing hypotheses will soon become depleted or ineffective because of gap expansions. The purpose of providing a greater amount of instantaneous manual annotation associated with increased data acquisition, while being prepared to address the possibility of having to make best use of purely human labour, creates a virtually insurmountable dilemma^8^. It is because this approach is totally dependent on well-trained professional biocurators who can analyse and extract categorized information from the published literature.

Text mining denotes the process of deriving high-quality information from text through the devising of patterns and trends. When properly processed by text mining methods, biomedical data may provide invaluable information to affect peoples’ cognizance of biomedical phenomena^9^. In 2005, most text mining tools were suited merely to a limited number of tasks^10^. With more yearly biomedical challenges and newly published databases such as i2b2, TREC Medical/CDS and BioNLP, biomedical text mining techniques have been driven to progress sharply. Hisschman *et al*. list the following four requirements for a biocurator text mining tool^11^: 1. Easy to use, install and maintain by the intended end user; 2. The tool need not be perfect, but it needs to complement the biocurator’s function; 3. Initial batch processing is necessary; and 4. The tool should provide linking of gene expression mentions of biological entities identified in the text with their referents identified in biological databases, then link them to the appropriate ontological terms.

Pathway analysis is an efficient method for gaining insight into the underlying biology of differentially expressed genes and proteins with less complexity and better explanatory power. KEGG PATHWAY^12^ collects manually drawn pathway maps integrating information from metabolism, genetic information processing, environmental information processing, cellular processes, organismal systems and human diseases. The workflow of biological pathway construction consists of four steps^13^ that serve as mining information resources, using pathway building tools, refinement, and leading to the desired specific annotated pathway, a process that can be very time-consuming and costly. Therefore, computational methods are needed to characterize the pathways automatically. Traditional *in silico* pathway annotation methods still rely on biologists to properly define features and guide feature selection during pathway prediction^14^, which creates yet another time- and cost-consuming step. Moreover, the results are highly dependent on the selected features, and if the features are not good enough, the achieved result may not be satisfying.

Intended for learning multiple levels of feature composition, models based on deep learning have become state-of-the-art methods in thriving research areas such as image recognition^15–17^, speech recognition^18–20^, language translation^6,21,22^, sentiment analysis^23–25^, image caption^26–28^ and so on. Deep learning is a powerful tool for discovering intricate structures in high-dimensional data^29^, which is common in biomedical informatics.

In this paper, faced with the task of sifting through the voluminous extant biomedical publications, we propose a Stacked Denoising Autoencoder Multi-Label Learning (SdaMLL) model to explore the effects, if any, gene multi-functions may have on cancer pathways in KEGG. To acquire more functions for each gene, we explore full texts of biomedical articles where more detailed methodologies, experimental results, critical discussions and interpretations can be found^30^. To the best of our knowledge, this is the first work applying a deep learning model^31^ to analyse gene multi-functions relevant to cancer pathways derived from full-text biomedical publications. In addition, the entire procedure proposed in this study does not require the involvement of a biologist to do a feature study about the data. Experimental results on eight KEGG cancer pathways reveal that SdaMLL is not only superior to classical multi-label learning models such as K-nearest neighbours and decision tree, but it can also achieve numerous gene functions related to important cancer pathways.

## Material and Methods

**Figure 1:**
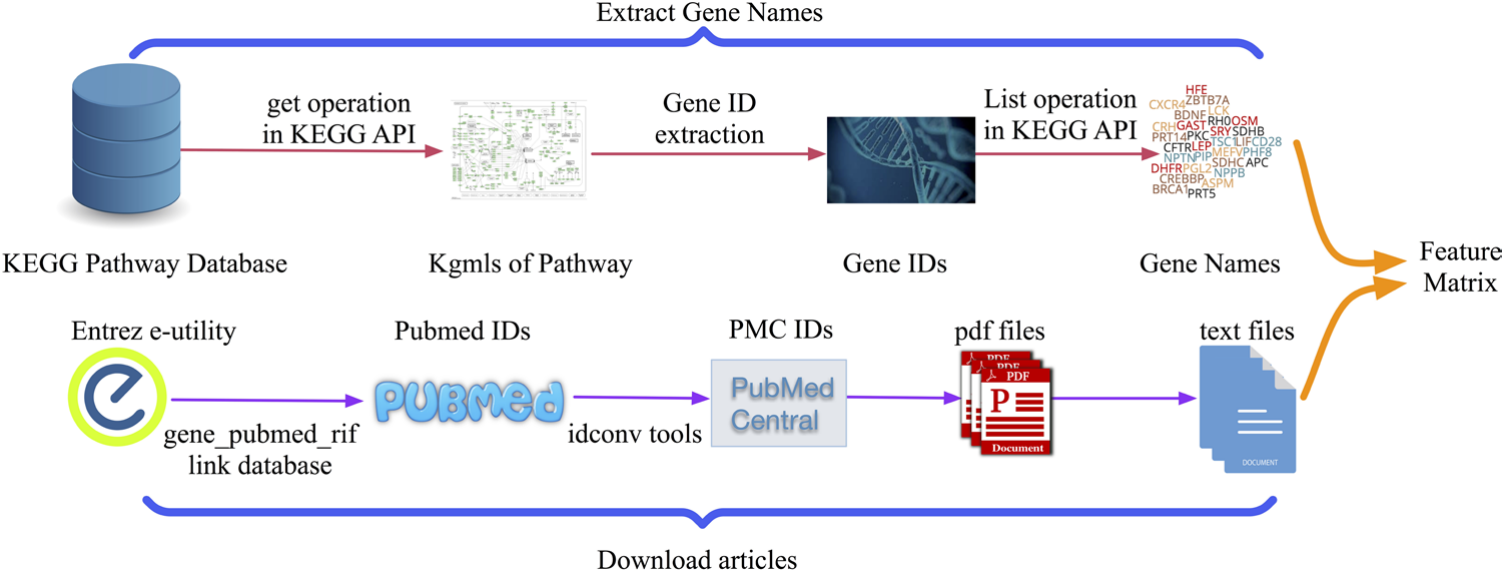
Feature matrix generation flowchart. The upper half illustrates the process for extracting all the gene names from the pathways in KEGG^12^ and the other half shows how we select articles that embed descriptions about gene function.

Faced with the challenge of reconciling a tremendous body of biomedical literature with its biological referents, we deem deep learning to be one of the most promising methods for fleshing out the relationship between them, and particularly in this paper, relationships with genes affecting cancer pathways. To predict multi-functions for each gene, we first generate a feature matrix following the routine in Fig. 1.

To generate the feature matrix, as shown in Fig. 1, on the one hand, we first extract all the gene IDs from a given KGML file. A KGML file provides information on reaction objects and their interactions annotated in the KEGG pathway plots, and the orthologous gene annotations from the KEGG GENES database. Widespread biomedical ambiguity is a well-known issue in bioinformatics. Therefore, we try to identify and tag all of the salient referents of a particular gene. Through the KEGG Entry list API, the most frequently used names for each gene in all the eight pathways are successfully gathered. On the other hand, with the help of Gene_pubmed_rif relation assembled in the ELink utility provided by NCBI, the PubMed id of all articles covering specific gene functions are fetched. Since a small portion of the articles are not open access, articles possessing both PubMed id and PMC id in a text file containing the list of all the downloadable pdfs via PMC FTP Service are left remaining. After downloading all 18930 pdf files, we extracted the required text content. The resulting matrix represents genes with term frequencies.

**Figure 2:**
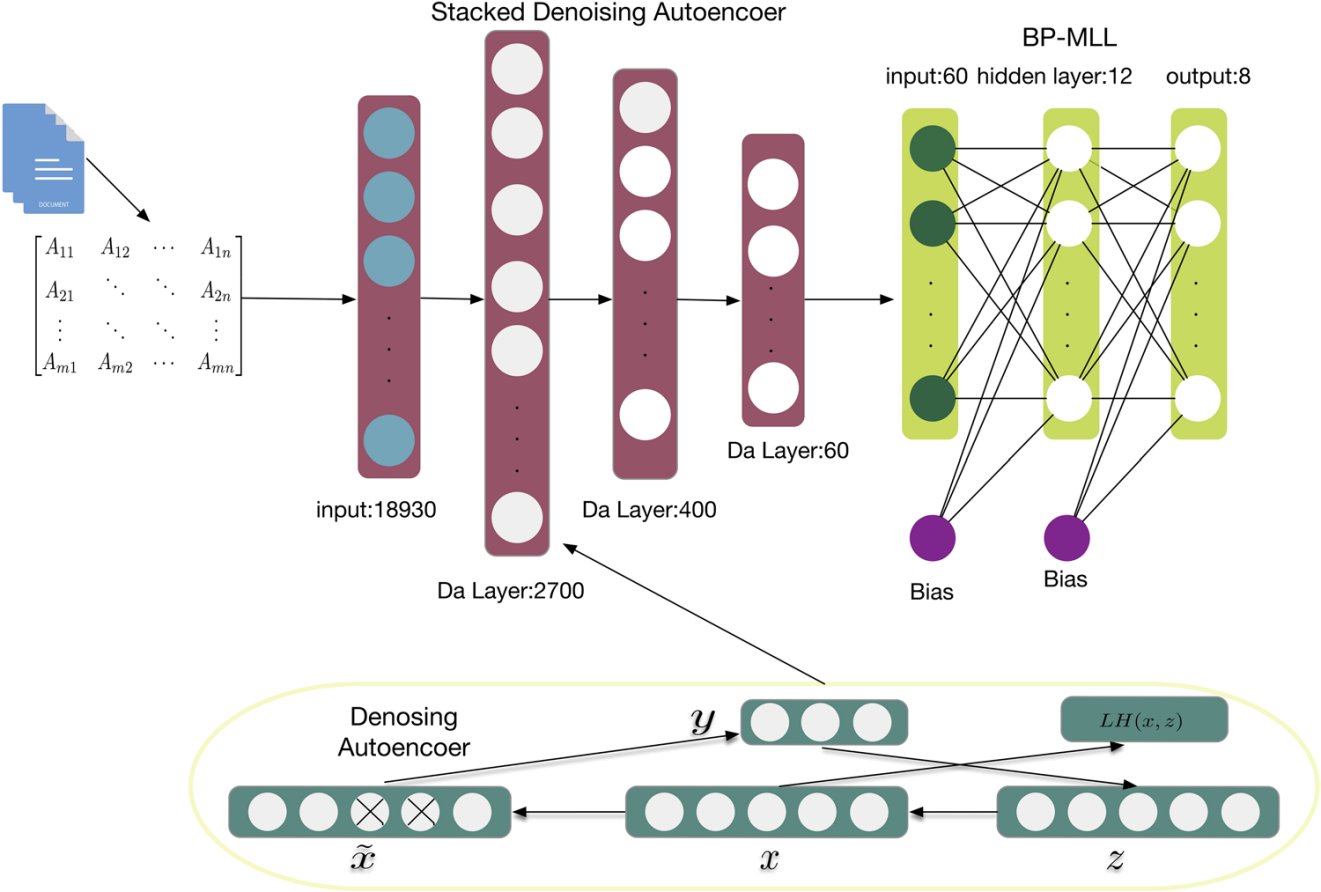
The architecture of SdaMLL. Each row of the feature matrix represents a gene. After being fed into the stacked denoising autoencoder, the original vector is tuned by removing various noises. The output of the autoencoder is then provided as the input to BP-MLL, predicting the gene assignment to the pathways.

After generating the feature matrix, the deep learning-based method SdaMLL can unveil gene multi-function. As shown in Fig. 2, SdaMLL consists of two modules: (a) Stacked denoising autoencoders (SdAs) serve as the representation learner, capturing the dependencies between dimensions in high dimensional distribution, and (b) the backpropagation for Multi-Label Learning (BP-MLL) focuses on finding the proper pathway label for each gene.

A traditional autoencoder involves encoding and decoding. Encoding maps the input *x* ∈ [0, 1]^*d*^ to a hidden representation *y* ∈ [0, 1]^*d*′^ through a deterministic mapping:

1 $${\bf{y}}={s}_{\theta }({\bf{Wx}}+{\bf{b}})$$

where *θ *= {**W**, **b**} and s denotes the sigmoid function. The code *y* is a latent representation of *x*, and it is then mapped back into *z*, which has the same shape as *x*. The reconstruction mapping process is formulated using a similar transformation:

2 $${\bf{z}}={s}_{\theta ^{\prime} }({\bf{W}}^{\prime} {\rm{y}}+{\bf{b}}{\boldsymbol{^{\prime} }})$$

where *θ*′ = {**W**, **b**′}. The autoencoder is constrained to have tied weights, which means **W **= **W**. The goal of autoencoder is to minimize the average reconstruction error:

3 $$\begin{array}{rcl}{\theta }^{\ast },{\theta ^{\prime} }^{\ast } & = & \mathop{{\rm{\arg }}\,{\rm{\min }}}\limits_{\theta ,\theta ^{\prime} }\frac{1}{n}\sum _{i\mathrm{=1}}^{n}L({{\bf{x}}}^{(i)},{{\bf{z}}}^{(i)})\\  & = & \mathop{{\rm{\arg }}\,{\rm{\min }}}\limits_{\theta ,\theta ^{\prime} }\frac{1}{n}\sum _{i=1}^{n}L({{\bf{x}}}^{(i)},{s}_{\theta ^{\prime} }({s}_{\theta }({{\bf{x}}}^{(i)})))\end{array}$$

For purposes of extending the hidden layer’s ability to discover more robust features and avoiding learning the identity simply, the denoising autoencoder is trained from a corrupted version of input. The denoising autoencoder is a stochastic version of an autoencoder, i.e., the initial input **x** is stochastically mapped to $\tilde{{\bf{x}}}$ with $\tilde{{\bf{x}}}\sim {q}_{{\mathscr{D}}}(\tilde{{\bf{x}}}|{\bf{x}})$. As shown in Fig. 2, for each input **x**, a fixed number *vd* of elements is randomly chosen to be reset as 0, leaving others unchanged. The corrupted version of the original input, $\tilde{{\bf{x}}}$, is then mapped to a hidden representation from which we reconstruct. The mapping and reconstruction processes are the same as those performed in a typical autoencoder. The goal of the denoising autoencoder is to minimize the reconstruction error ${L}_{H}({\bf{x}},{\bf{z}})={\mathbb{H}}({ {\mathcal B} }_{{\bf{x}}}|{ {\mathcal B} }_{{\bf{z}}})$ over the training set as well. A multi-layer denoising autoencoder could be constructed by combining multiple single-layer denoising autoencoders and connecting the output of the previous layer to the input of the next one. Each layer may be tuned by an unsupervised pre-training. Once the first k layers are trained, we can train the (*k *+ 1)^*th*^ layer because we can now compute the code or latent feature representation from the layer below.

The upper right part of Fig. 2 illustrates the framework of BP-MLL, which is a typical feed-forward artificial neural network modified for multi-label learning. BP-MLL solves the problem with two notable modifications: (a) a specifically designed error function and (b) a revision made according to the classical learning algorithm. It outperforms several existing methods in functional genomics and text categorization^32^. Let $\chi ={{\mathbb{R}}}^{d}$ denote the instance domain and *γ* = 1, 2, …, *N*, indicates the set of all the class labels. *m* indicates multi-label instances, and each *Y*_*i*_ in {(*x*_1_, *Y*_1_), (*x*_2_, *Y*_2_),…, (*x*_*n*_, *Y*_*n*_)} may contain several labels. The global error function is rewritten as

4 $$E=\sum _{i=1}^{m}{E}_{i}=\sum _{i\mathrm{=1}}^{m}\frac{1}{|{Y}_{i}||{\bar{Y}}_{i}|}\sum _{(k,l)\in {Y}_{i}\times {\bar{Y}}_{i}}\exp (-({q}_{k}^{i}-{q}_{l}^{i}))$$

where for the *i*-th training example (*x*_*i*_, *Y*_*i*_), the error term is $\frac{1}{|{Y}_{i}||{\bar{Y}}_{i}|}\sum _{(k,l)\in {Y}_{i}\times {\bar{Y}}_{i}}exp(-({q}_{k}^{i}-{q}_{l}^{i}))$. The complementary set of *Y*_*i*_ in *γ* is ${\bar{Y}}_{i}$ and |·| is the cardinality of a set. The difference between the outputs of BP-MLL on one label belonging to *x*_*i*_(*k* ∈ *Y*_*i*_) and one label not belonging to it $(l\in {\bar{Y}}_{i})$ is measured by ${c}_{k}^{i}-{c}_{l}^{i}$. The performance gets better when the difference increases. Gradient descent strategy is applied to reduce the error:

5 $$\begin{array}{rcl}{{\rm{\Delta }}}_{{w}_{sj}} & = & -\,\alpha \frac{\partial {E}_{i}}{\partial {w}_{sj}}=-\alpha \frac{\partial {E}_{i}}{\partial net{c}_{j}}\frac{\partial net{c}_{j}}{\partial {w}_{sj}}\\  & = & \alpha {d}_{j}[\frac{\delta (\sum _{s\mathrm{=1}}^{M}{b}_{s}{w}_{sj})}{\partial {w}_{sj}}]=\alpha {d}_{j}{b}_{s}\end{array}$$

6 $$\begin{array}{rcl}{{\rm{\Delta }}}_{{v}_{hs}} & = & -\,\alpha \frac{\partial {E}_{i}}{\partial {v}_{hs}}=-\alpha \frac{\partial {E}_{i}}{\partial net{b}_{s}}\frac{\partial net{b}_{s}}{\partial {v}_{hs}}\\  & = & \alpha {e}_{s}[\frac{\delta (\sum _{h\mathrm{=1}}^{d}{a}_{h}{v}_{hs})}{\partial {v}_{hs}}]=\alpha {e}_{s}{a}_{h}\end{array}$$

the bias are changed according to:

7 $${\rm{\Delta }}{\theta }_{j}=\alpha {d}_{j};\,\Delta {\gamma }_{s}=\alpha e$$

where *α* is the learning rate and its range is (0.0, 1.0). When training the BP-MLL, the training instances are fed to the network one by one. For each multi-labeled instances (*x*_*i*_,*Y*_*i*_), the weights are updated according to Eqs 5 and Eqs 7. When global error *E* doesn’t decrease or the training epochs hits a threshold, it stops training.

## Results and Discussion

The data used in our experiment is downloaded from the two databases KEGG PATHWAY and PubMed Central. Term frequencies are computed for all the words appearing in pathway-related articles retrieved from PubMed Central. To construct the feature matrix, we collect the gene names for all 1144 genes and 18930 articles closely related to these genes. The feature matrix is constructed using all the genes listed in the pathway map overviews and gene-related full-text articles fetched from Pubmed. For purposes of validating the generalization ability of the proposed method, 10-fold cross-validation was conducted while only being able to use a limited number of genes extracted from the existing KEGG pathways. Regarding the pathways, we selected the cancer pathways according to the following widely influential references. Douglas *et al*. summarized six capabilities acquired by most forms of cancer, and they added two more cancer traits in 2011^33,34^. Parallel pathways take part in the tumourigenesis process^35–37^. During the process, traits of cancers can be arranged in multiple permutations, which means cell transformation occurs. Semir Beyaz et. al have proposed that uncovering how PPAR-*δ* mediates tumourigenesis in diverse tissues and cell types in response to diet may enhance clinical utility^38^. Niall *et al*. regard targeting DNA repair and repair-deficient tumours as new avenues for treating advanced disease in the future^39^.

We compare SdaMLL with the following methods: K-nearest neighbours (KNN), decision trees (DTs)^40^ and Backpropagation for Multi-Label Learning (BP-MLL)^32^. KNN is one of the most commonly used machine learning methods. The goal of the nearest neighbour method is to find a pre-determined number of training samples closest in distance to the new point and predict the labels among these samples. DTs is a non-parametric supervised learning classification method that aims at learning simple decision rules derived from original data and predicting results with these rules. BP-MLL is derived from the backpropagation^41^ algorithm via applying an error function acquiring the characteristics of multi-label learning. This is the first time this term has been employed in functional genomics and text categorization.

In our experiment, we adopted three different metrics for multi-label learning proposed by Schapire et. al, which are coverage, ranking loss, and average precision^42^. Coverage evaluates the performance of a system for the top-ranked label, i.e., how dire is the need to go further down the set of labels to cover all the correct labels of each instance, on average. The performance is improved when the value decreases. For a set of labeled documents *S* = 〈(*x*_1_, *Y*_1_), …, (*x*_*m*_, *Y*_*m*_)〉, the coverage is

8 $$coverag{e}_{S}(H)=\frac{1}{m}\sum _{i\mathrm{=1}}^{m}\mathop{{\rm{\max }}\,}\limits_{\ell \in {Y}_{i}}ran{k}_{f}({x}_{i},\ell )-1$$

Ranking loss evaluates the fraction of label pairs in which an irrelevant label is ranked higher than a relevant label. When the *rloss*(*H*) = 0, the performance is perfect. For a set of labeled documents *S* = 〈(*x*_1_, *Y*_1_),…, (*x*_*m*_, *Y*_*m*_)〉, ranking loss is defined as

9 $$rloss(H)=\frac{1}{m}\sum _{i\mathrm{=1}}^{m}\frac{|{D}_{i}|}{|{Y}_{i}||{\bar{Y}}_{i}|}$$

where $\bar{Y}$ is the complementary set of *Y* in *γ* and ${D}_{i}=\{({y}_{1},{y}_{2})|\,f({x}_{i},{y}_{1})\le f({x}_{i},{y}_{2}),({y}_{1},{y}_{2})\in {Y}_{i}\times {\bar{Y}}_{i}\}$

The average precision evaluates the average faction of labels ranked above a particular label *y*∈*Y*, which actually are in *Y*. The performance is better when the value of average precision is bigger.

10 $$avg\,pre{s}_{S}(H)=\frac{1}{m}\sum _{i\mathrm{=1}}^{m}\frac{1}{|{Y}_{i}|}\sum _{\ell \in {Y}_{i}}\frac{|\{{\ell }^{\text{'}}\in {Y}_{i}|ran{k}_{f}({x}_{i},{\ell }^{\text{'}})\le ran{k}_{f}({x}_{i},\ell )\}|}{ran{k}_{f}(x,\ell )}$$

1114 distinct genes are distributed in 8 pathways, which leads to a relatively small amount of data. Under such circumstances, we try to check the generalization ability of SdaMLL and other methods with 10-fold cross-validation. During the experiments, the dataset is randomly divided into 10 separate subsets with only one subset serving as validation in each assessment.

**Figure 3:**
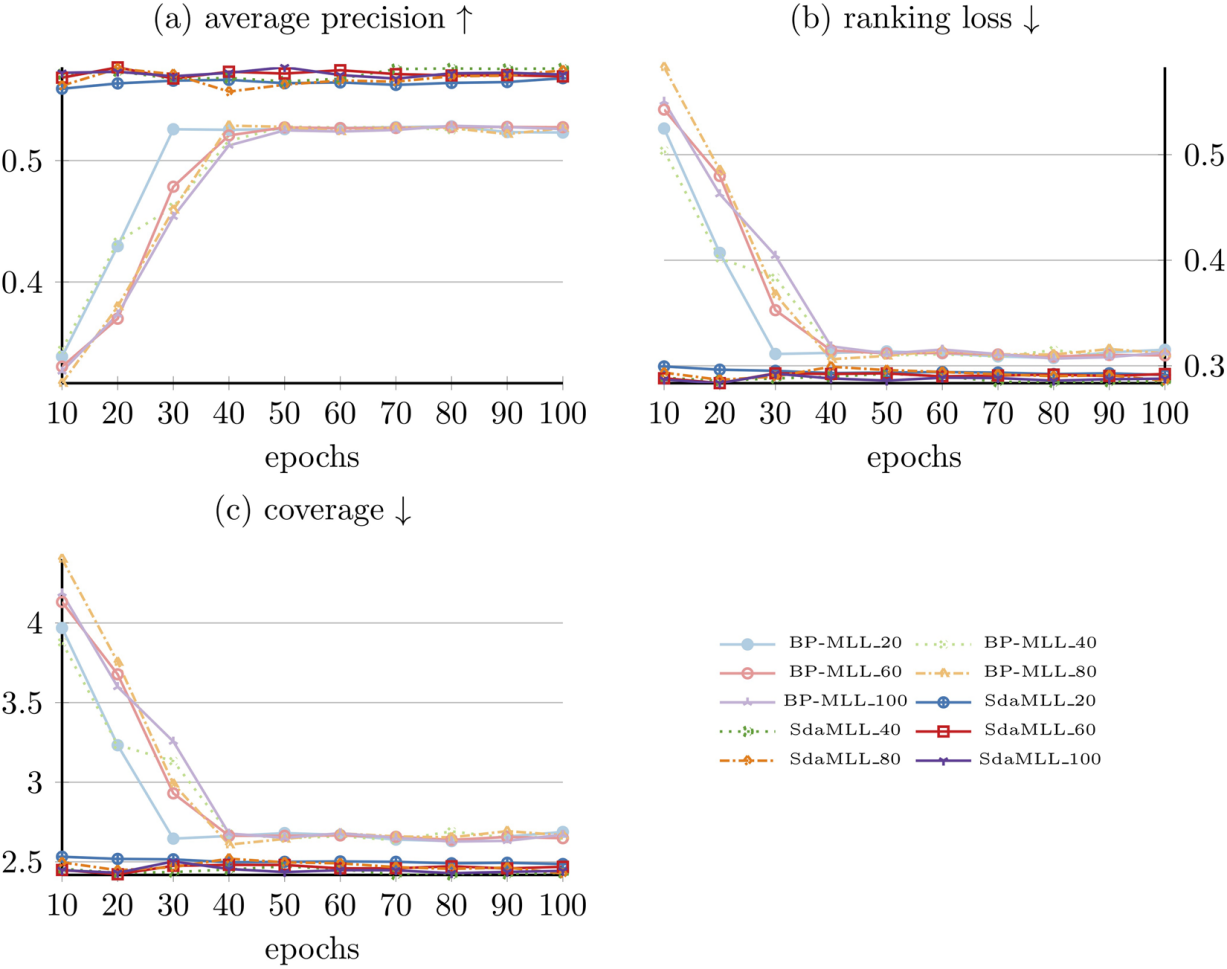
The Comparison between BP-MLL and SdaMLL. ↑ denotes that the model’s performance is better when the metrics are larger and vice versa.

Results for average precision can be found in the second column in Table 1, and Fig. 3(a) elucidates a detailed comparison between SdaMLL and BP-MLL. SdaMLL achieves the best performance among the 4 algorithms. SdaMLL’s average precision is 0.577, while KNN’s average precision is 0.333. Compared to KNN, SdaMLL’s is 73.27% higher. BP-MLL’s average precision is the second highest when confronted with the other algorithms. Nevertheless, to reach such a high average precision, BP-MLL requires 40 more training epochs than SdaMLL. In contrast, SdaMLL converges with fewer than 10 training epochs.

**Table 1:** Experiments results for all multi-label classification algorithm. Results of BP-MLL and SdaMLL with highest average precision are selected.

	Cverage precision	Ranking loss	Coverage
KNN	0.333	0.662	95.25
Decision trees	0.385	0.644	100.763
SdaMLL	**0.577**	**0.286**	**2.436**
BP-MLL	0.529	0.306	2.608

In the third column (Ranking Loss) of Table 1, we can see the value of ranking loss, and a detailed comparison between BP-MLL and SdaMLL appears in Fig. 3(b). SdaMLL’s ranking loss is 0.286, which is 0.376 lower than KNN, and which represents the best performance among the 4 algorithms. The results are uniformly comparable to the values in the second column; KNN and decision trees get similar results; and SdaMLL and BP-MLL’s ranking loss values are alike. When BP-MLL is compared with SdaMLL, the difference becomes increasingly clear. SdaMLL, again, converges much faster than BP-MLL.

The forth column (Coverage) of Table 1 has the coverage values. Figure 3(c) shows a comparison between BP-MLL and SdaMLL. SdaMLL achieves the best performance and remains stable during the training no matter how variable the number of epochs is. The coverage of SdaMLL, 2.436, is relatively low, constituting a significant signal that SdaMLL will not go deeply into the set of labels when determining correct labels for particular instances. In the meantime, while the result of BP-MLL’s coverage is approximately low, it is 0.172 higher than SdaMLL. However, the performance disparity between SdaMLL and decision trees is very spectacular. Specifically, decision tree’s convergence is virtually 40 times greater than that of SdaMLL.

For the time consumption, it costs 153.07 s to train the BP-MLL model per epoch while the time of training the SdaMLL model is averaged to 38.46 s per epoch. The training time for KNN and decision tree is 0.42 s and 6.63 s. All the prediction time of KNN and decision tree is 12.6 s and 16ms, however, BP-MLL and SdaMLL is 7.16 s and 4.56 s. From the prediction time comparison, it can be seen that SdaMLL needs more time than decision trees, however, it is faster than KNN.

Based on the results of the aforementioned measurements, SdaMLL outperforms BP-MLL in three respects: (1) SdaMLL converges far more quickly than BP-MLL, requiring 30 fewer training epochs on average; (2) On all of the 3 metrics, SdaMLL achieves better results than BP-MLL; and, (3) Original BP-MLL is time-consuming, while SdaMLL finishes in less time. Although the process of constructing the feature matrix is complicated, when one considers the difference between the training data distribution and the actual data distribution, the training data distribution is probably corrupted. Additionally, each instance is a 1 × 18930 vector, and most of the bits in a particular vector are zero, creating a data sparsity problem in which the data are extremely hard to interpret. Stacked denoising autoencoders, on the one hand, try to capture intermediate representation, which is robust compared to a partial corruption of the input pattern. In addition, by initializing the weights effectively, the architecture of the autoencoder is able to generate low-dimensional codes that surpass common dimension reduction tools such as principal component analysis^43^ and independent component analysis^44^ (ICA).

Apart from the quantity improvements, we can also ascertain the biological results. As mentioned in the dataset description, all the collected articles indicate particular functions of genes, which can serve as evidence for judging whether the gene serves as part of a pathway. From this prediction, we also found some articles that can solidly back up the correlation between genes and pathways. First, tumour Protein P53 (TP53) encodes a tumour suppressor protein containing transcriptional activation, DNA binding, and oligomerization domains. The encoded protein responds to diverse cellular stresses to regulate expression of target genes, thereby inducing cell cycle arrest, apoptosis, senescence, DNA repair, or changes in metabolism. Saha *et al*. demonstrated a possible role for the candidate tumour suppressor ING genes in the biology of EBV-associated cancer^45^, and the N-terminal domain of EBNA3C residues 129 to 200 was previously demonstrated to associate with p53. From these clues, we may regard p53 as part of the pathway describing pathways in cancer (hsa05200).

Moreover, FUS or FUS/TLS (Fused in sarcoma/translocated in liposarcoma) is a multifunctional RNA/DNA-binding protein that is pathologically associated with cancer and neurodegeneration. It encodes a multifunctional protein component of the heterogeneous nuclear ribonucleoprotein (hnRNP) complex. From an article^46^, we can conclude that FUS-DDIT3 deregulates some NF-kappaB-controlled genes through interaction with NFKBIZ. FUS related genes are involved in tumour type-specific fusion oncogenes in human malignancies, which indicates that FUS is probably part of transcriptional misregulation in cancer (hsa:05202).

In addition, P27 or Kip1 (Cyclin-Dependent Kinase Inhibitor 1B) encodes a cyclin-dependent kinase inhibitor, in which mutations are associated with multiple endocrine neoplasia type IV. The encoded protein binds to and prevents the activation of cyclin E-CDK2 or cyclin D-CDK4 complexes, and thus controls cell cycle progression at G1. PKB/Akt mediates cell-cycle progression by phosphorylation of P27 (Kip1) at threonine 157, and modulation of its cellular localization indicates that Akt may contribute to tumour-cell proliferation by phosphorylation and cytosolic retention of p27, thus relieving CDK2 from p27-induced inhibition^47^. Obviously, it is also a hint that p27 becomes a member of viral carcinogenesis (hsa:05203). Other foundlings such as CCNA2, EGR2, and MAPKAPK2 belong to viruses or encode cancer pathways; CCND1, NRAS, and SDC1 all contribute to the proteoglycans pathway; AKT1, MET, TP53 and MAPK1 are adaptations of cellular metabolism pathway elements.

These three samples are the solid proof for our biology discovery. More interesting examples (270 new functions) are listed at https://www.keaml.cn/gpvisual.

**Figure 4:**
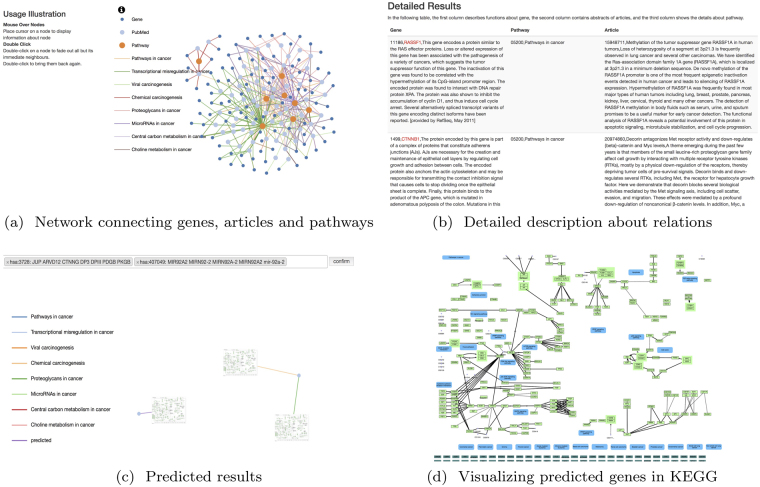
Illustration of new gene functions. (**a**) Network connecting genes, articles and pathways. The yellow points are the pathways, light blue points are the PubMed manuscripts and blue points are functional genes. (**b**) Detailed description about relations. All the relationships are listed as triple-items {gene, pathway, article}. (**c**) Predicted results. The illustration of our predicted results about gene functions. (**d**) Visualizing predicted genes in KEGG. The completion results for KEGG pathways^12^ are listed at the bottom of the web page.

We also provide a visualization tool for multi-function genes in cancer pathways. Based on the text mining results, we find it is common for a gene to get involved in multiple pathways. For the sake of unveiling the relationship between genes and pathways, we try to visualize a network of genes, articles, and pathways. In Fig. 4(a), we can observe a primitive network illustrating help edges connecting genes and pathways. If users click on the info button, the helper information containing a definition for each entity and lines in the network will either appear or hide. In Fig. 4(b), specifics about each link can be found in the details tab. Users can input the desired name or ID of specific genes to dig out relations between those genes and pathways, as depicted in Fig. 4(c). Meanwhile, thumbnails of predicted pathways will pop out. If users click on the thumbnails, the browser will jump to a detailed description of that pathway, as shown in Fig. 4(d). In the new tab, predicted results are listed at the bottom of the web page.

## Conclusion and Future Work

In this paper, we have proposed a novel multi-label gene function annotation model based on a deep learning strategy, namely, SdaMLL, for gene multi-function discovery. This model takes advantage of both effective dimension reduction and multi-label classification on account of Stacked denoising autoencoders. Compared to BP-MLL, SdaMLL converges much faster in terms of the number of training epochs. In addition, during the experiments, we try to reduce the dimension from 18900 to 200, which helps to shorten the training time tremendously. From the results, we can conclude that SdaMLL is a state-of-the-art algorithm for finishing the task at hand. In addition, we provide a website for researchers to inspect relationships between genes and articles.

This study demonstrated how the proposed method performed based on the data of eight pathways and generated a feature matrix containing genes existing in these pathways. Better annotation performance may be anticipated if more information from other pathways is integrated into the model in the future.
