# Author Correction: Multi-label Deep Learning for Gene Function Annotation in Cancer Pathways

**DOI:** 10.1038/s41598-018-27349-6

**Published:** 2018-06-07

**Authors:** Renchu Guan, Xu Wang, Mary Qu Yang, Yu Zhang, Fengfeng Zhou, Chen Yang, Yanchun Liang

**Affiliations:** 1 Key Laboratory for Symbol Computation and Knowledge Engineering of National Education Ministry, College of Computer Science and Technology, Jilin University, Changchun, 130012 China; 2 Zhuhai Laboratory of Key Laboratory of Symbolic Computation and Knowledge Engineering of Ministry of Education, Zhuhai College of Jilin University, Zhuhai, 519041 China; 3 MidSouth Bioinformatics Center and Joint Bioinformatics Ph.D. Program of University of Arkansas at Little Rock and Univ. of Arkansas Medical Sciences, Little Rock, AR 72204 USA; 4 Institute of Information Engineering, Chinese Academy of Sciences School of Cyber Security, University of Chinese Academy of Sciences, Beijing, 100093 China; 5 College of Earth Sciences, Jilin University, Changchun, 130061 China

Correction to: *Scientific Reports* 10.1038/s41598-017-17842-9, published online 10 January 2018

The Acknowledgements section of this Article is incomplete.

“The authors are grateful for the support of the National Natural Science Foundation of China (No.61572228, No.61472158, No.61300147, No.61602207), United States National Institutes of Health (NIH) Academic Research Enhancement Award (No.1R15GM114739), National Institute of General Medical Sciences (NIH/NIGMS) (No.5P20GM103429), United States Food and Drug Administration (FDA) (No.HHSF223201510172C), the Science Technology Development Project from Jilin Province (No.20160101247JC), Zhuhai Premier-Discipline Enhancement Scheme and Guangdong Premier Key-Discipline Enhancement Scheme. This work was partially supported by the Strategic Priority Research Program of the Chinese Academy of Sciences (XDB13040400) and a start-up grant from the Jilin University. We thank KEGG group for supporting pathway data.”

should read:

“The authors are grateful for the support of the National Natural Science Foundation of China (No.61572228, No.61472158, No.61300147, No.61602207), United States National Institutes of Health (NIH) Academic Research Enhancement Award (No.1R15GM114739), National Institute of General Medical Sciences (NIH/NIGMS) (No.5P20GM103429), the Science Technology Development Project from Jilin Province (No.20160101247JC), Zhuhai Premier-Discipline Enhancement Scheme and Guangdong Premier Key-Discipline Enhancement Scheme. This work was partially supported by the United States Food and Drug Administration (FDA), contract No. HHSF223201510172C and HHSF223201610111C. However, the information contained herein represents the position of the author(s) and not necessarily that of the NIH and FDA. This work was partially supported by the Strategic Priority Research Program of the Chinese Academy of Sciences (XDB13040400) and a start-up grant from the Jilin University. We thank KEGG group for supporting pathway data.”

